# Prevalence and predictors of overweight and obesity among Cameroonian women in a national survey and relationships with waist circumference and inflammation in Yaoundé and Douala

**DOI:** 10.1111/mcn.12648

**Published:** 2018-07-26

**Authors:** Reina Engle‐Stone, Martin Nankap, Alex O. Ndjebayi, Avital Friedman, Ann Tarini, Kenneth H. Brown, Lucia Kaiser

**Affiliations:** ^1^ Department of Nutrition University of California Davis California; ^2^ Helen Keller International, Cameroon Yaoundé Cameroon; ^3^ Helen Keller International New York New York; ^4^ Bill & Melinda Gates Foundation Seattle Washington

**Keywords:** abdominal obesity, Africa, maternal obesity, nutrition transition, overweight

## Abstract

Information on the distribution and predictors of obesity in Africa is needed to identify populations at risk and explore intervention options. Our objectives were to (a) examine the prevalence and geographic distribution of overweight and obesity among Cameroonian women; (b) evaluate change in anthropometric indicators among urban women between 2009 and 2012; (c) examine associations between household and individual characteristics and overweight and obesity; and (d) examine relationships between body mass index (BMI), abdominal obesity, and inflammation. We analysed data from a nationally representative survey conducted in 3 geographic strata (North, South, and Yaoundé/Douala) in Cameroon in 2009 and a survey in Yaoundé/Douala in 2012. Participants selected for this analysis were nonpregnant women, ages 15–49 years (*n* = 704 in 2009; *n* = 243 in 2012). In 2009, ~8% of women were underweight (BMI < 18.5) and 32% overweight or obese (BMI ≥ 25.0). Underweight was most common in the North (19%) and overweight and obesity in the South (40%) and Yaoundé/Douala (49%). Prevalence of BMI ≥ 25.0 in Yaoundé/Douala did not differ in 2012 compared with 2009 (55.5% vs. 48.7%; *P* = 0.16). Residence in urban areas, greater maternal age, and TV ownership were independently related to overweight and obesity in national and stratified analyses. In Yaoundé/Douala in 2012, 48% (waist‐to‐hip ratio > 0.85) to 73% (waist circumference > 80 cm) had abdominal obesity. Body mass index was positively associated with abdominal obesity and inflammation. Though causal inferences cannot be drawn, these findings indicate population subgroups at greatest risk for overweight and associated health consequences in Cameroon.

**Key messages**

- Overweight and obesity affect one third of women in Cameroon and are particularly common in the cities of Yaoundé and Douala and other urban areas.
- Overweight and obesity were more common among older and more educated women, women who consumed sweetened beverages, and women in households with TV ownership and greater socio‐economic status.
- BMI was positively associated with markers of abdominal adiposity and inflammation, suggesting increased cardiometabolic risk.
- Underweight and micronutrient deficiencies are still prevalent in Cameroon, particularly the North, so interventions to prevent malnutrition in all forms are needed.

## 1 INTRODUCTION

The prevalence of overweight and obesity is increasing, even in low‐income countries (NCD Risk Factor Collaboration (NCD‐RisC), 2016). Projections are for an increased prevalence of overweight and obesity, from 1.3 billion people affected in 2005 to 3.28 billion in 2030; currently 57 of 129 countries have high prevalence of both underweight and overweight (Global Panel on Agriculture and Food Systems for Nutrition, 2016). The growing worldwide problem of overweight, obesity, and diet‐related chronic diseases in the face of persistent poor‐quality diets is thought to be driven by urbanization and globalization. Food system changes may still be able to prevent dire outcomes in countries in earlier stages of nutrition transition.

In West Africa, most countries are in the early stages of transitioning from undernutrition to overnutrition, though some (Ghana, Cape Verde, and Senegal) are in later stages (Bosu, 2015). For example, Ghana has experienced dramatic increases in maternal overweight/obesity from 12.8% in 1993 to 29.2% in 2008. The nutrition transition in this region is associated with an increase in other risk factors, for example, hypertension in Cameroon (Bosu, 2015; Fezeu, Kengne, Balkau, Awah, & Mbanya, 2010). Diabetes prevalence is also increasing in some areas within countries, particularly urban Ghana and Senegal (Bosu, 2015), and incidence is projected to double between 2000 and 2030 (Haggblade et al., 2016). In a systematic review of African studies conducted between 1972 and 2011, maternal obesity (body mass index [kg/m^2^] ≥ 30, based on pre‐pregnancy or first trimester measurements) occurred in 9–17.9% of pregnancies and was associated with increased risk of C‐section birth deliveries, macrosomia, gestational diabetes, pregnancy‐induced hypertension, and preeclampsia but decreased risk of maternal anaemia and low birthweight (Onubi, Marais, Aucott, Okonofua, & Poobalan, 2016). Although information is sparse, available data suggest that the burden of overweight is greatest in urban areas and among wealthier, older women (Jones, Acharya, & Galway, 2016; Kandala & Stranges, 2014). In Cameroon, urban residents reported lower levels of physical activity than their rural counterparts, but the relationship between physical inactivity and obesity was more consistent for men than women (Sobngwi et al., 2002). A cross‐sectional study among adults of African origin from Cameroon, Jamaica, and the United Kingdom also found gender to influence the relationship of dietary intakes to overweight or obesity (Jackson et al., 2007).

Since prevalence of maternal overweight and obesity varies geographically within and across countries in sub‐Saharan Africa, more research is also needed in this region on the prevalence and distribution of these conditions. In addition, evaluating the relationship of BMI to other risk factors, including abdominal obesity, as measured by waist circumference, and inflammation, may provide insight regarding the risks of overweight in this context. In a cross‐sectional study of community‐based samples from urban and rural Cameroon and France, the relationship of waist circumference to blood pressure, blood lipids, fasting blood glucose, and other risk factors differed by ethnicity/race, gender, and urbanization (3). Elsewhere in Africa, Traissac et al. found abdominal obesity to be more prevalent and variable than overall obesity based on BMI and highlighted a need for more research among women living in a nutrition transition context (Traissac et al., 2015).

Timely, national studies of maternal weight status and child growth are urgently needed to guide policies and strategies to prevent nutrition‐related chronic diseases within countries and regions. Many studies in Africa still focus on exclusively on undernutrition, despite the emerging problem of maternal obesity and overweight within this region. This paper uses data from a 2009 national survey and a 2012 regional survey in Cameroon, conducted for the purpose of designing and evaluating a food fortification program. In this paper, the objectives were to (a) examine the prevalence and geographic distribution of overweight and obesity among women of reproductive age; (b) evaluate the change in anthropometric indicators between 2009 and 2012 in two major cities (Yaoundé and Douala); (c) examine the associations between household and individual characteristics and overweight and obesity among women; and (d) assess the prevalence of abdominal obesity (elevated waist circumference) and relationships between BMI and abdominal obesity and inflammation among women in urban areas.

## 2 METHODS

### 2.1 Setting

Cameroon is classified by the World Bank as a lower middle income country, with slightly over half the population living in urban areas (54% in 2015) and a gross domestic product per capita (purchasing power parity) estimated at $2,829 in 2014 (Global Nutrition Report, 2015). There has been limited progress on indicators related to health and undernutrition over the past decade: The under‐5 mortality rate was 95 per 1,000 in 2012, ranked 21st globally, and stunting prevalence was 33% in 2011 (Global Nutrition Report, 2015). Previous work revealed a high prevalence of anaemia and micronutrient deficiencies among women and children (Engle‐Stone, Ndjebayi, Nankap, & Brown, 2012), although large‐scale fortification appears to have reduced this risk for selected micronutrients (Engle‐Stone et al., 2017). At the same time, concerns about overweight and associated chronic disease risk in Cameroon have arisen. For example, a 2013 survey using the WHO STEPWISE approach to Surveillance (STEPS) methodology reported an age‐standardized prevalence of 29.7% for hypertension among the 15,470 adult participants from urban areas (Kingue et al., 2015).

This paper presents data from a nationally representative survey conducted in Oct–Dec, 2009, and a regional survey conducted in Oct–Nov, 2012. Detailed descriptions of the methods and primary results from these surveys have been reported elsewhere (Engle‐Stone et al., 2012; Helen Keller International Cameroun, Ministère de la Sante Publique du Cameroun, & UNICEF, 2011).

### 2.2 Sampling design and participants

#### 2.2.1 National survey

The 2009 survey was designed to be representative at the national level, and for each of three strata: South (the 7 southern administrative regions, except the cities of Yaoundé and Douala), North (the 3 northern administrative regions: North, Far North, and Adamawa), and Yaoundé/Douala (the two largest metropolitan areas, comprising ~22% of the total population) (Engle‐Stone et al., 2012). Thus, the South (representing ~45% of the national population) and North (~33% of total population) included both urban and rural areas. Within each stratum, 30 clusters were selected using probability proportionate to size sampling. Next, 10 households per cluster were selected by first identifying a random start point within the cluster and then conducting systematic sampling of nearby households.

Households were eligible for the survey if there was a child 12–59 months of age and a woman 15–49 years of age who was the child's primary female caregiver. If there were multiple children in the target age group in the household, a child was selected at random. Households were excluded if the woman and child had not lived in the household for at least 1 month, or if either individual had experienced severe fever, diarrhoea with dehydration, or other severe illness during the 3 days prior to data collection. Women provided informed oral consent for themselves and the index child to participate. The study was approved by the Institutional Review Board of the University of California, Davis, and the Cameroon National Ethics Committee.

#### 2.2.2 Regional survey in Yaoundé/Douala

The 2012 survey was conducted to examine the impact of large‐scale food fortification on micronutrient status (Engle‐Stone et al., 2017). The sampling framework was confined to Yaoundé and Douala due to resource limitations and greater expected availability of fortified foods compared with rural areas. To improve statistical power to detect changes over time, the 2012 survey was conducted in the same clusters that were selected in 2009, using the same methodology to select households (although the participating households differed between the two surveys). The surveys were conducted in the same season of the year to minimize the impact of seasonal variation on results. The 2012 survey protocol was approved by the Institutional Review Board of the University of California, Davis, and the Cameroon Ministry of Public Health (during a period of reorganization in which the National Ethics Committee was not reviewing protocols).

For both surveys, only women who reported that they were not currently pregnant and for whom anthropometric data were available were included in the current analysis.

### 2.3 Data collection

#### 2.3.1 Questionnaires, dietary data

Interviewers administered questionnaires to the index woman to collect information on variables relating to household socio‐economic status (SES), her exposure to media (originally for the purpose of assessing potential outlets for promotion of fortified foods), and frequency of intake of selected foods by the woman and child during the previous week. Pregnancy was determined by self‐report. Interviewers were female, and most had some college education. All interviewers were multilingual (spoke French, English, and at least one other local language) and completed a 1‐week study training on research ethics, interviewing techniques, and study procedures and instruments.

Within a questionnaire module on exposure to media, participants were asked how many days in the past 7 days they watched television and how many minutes they watched television on the last day they watched television (Helen Keller International Cameroun et al., 2011).

A food frequency questionnaire (FFQ), based on the Fortification Rapid Assessment Tool (Micronutrient Initiative & PATH Canada, 2003), was administered to collect information on selected foods or dishes prepared with any of four potentially fortifiable foods: refined cooking oil, wheat flour, sugar, or bouillon cube (Engle‐Stone et al., 2012). For each item, participants were asked how many days in the previous week they consumed the item, and how many times per day the item was consumed on the last day on which it was consumed. As indicators of processed snack food intake, the analysis described below included the mother's frequency of consuming (a) packaged biscuits (sweet and savoury); (b) sweets (hard candy, chocolate, and ice cream); (c) sweetened beverages (carbonated beverages, fruit‐flavoured juice, and popsicles); and (d) *beignets* (fried dough), which are prepared by local vendors and commonly consumed for breakfast or as a snack.

The original Fortification Rapid Assessment Tool questionnaire was field tested in multiple settings, but not formally validated. However, the adapted questionnaire used in this setting provided data consistent with the results of 24‐hr dietary recalls (Engle‐Stone et al., 2012). For example, both methods indicated almost ubiquitous consumption of bouillon cube (>90% of respondents consumed in the past day (24‐hr recall) and past week [FFQ]) and more frequent consumption of refined vegetable oil and wheat flour in Yaoundé/Douala compared with the South and North.

#### 2.3.2 Anthropometry

In both surveys, women were asked to remove shoes, hats, and any heavy outer clothing (jackets, etc.) prior to anthropometric measurements. Measurements were conducted by anthropometrists who completed study‐specific training and standardization exercises prior to the study. Height was measured in duplicate to the nearest 0.1 cm with a portable stadiometer (Seca Leicester Portable Height Measure, Seca Weighing and Measuring Systems), and weight was measured in duplicate to the nearest 0.1 kg with an electronic scale (Seca 899, Seca Weighing and Measuring Systems). For women with hairstyles that could not be compressed to measure the height of the crown of the head, the height of the hair was estimated and subtracted from the final measurement. If the difference between the two height or weight measurements was greater than 0.5 cm or 0.5 kg, respectively, a third measurement was taken and the closest two measures recorded. The mean difference in repeat measurements was 0.03 cm and 0.01 kg in 2009 and 0.04 cm and 0.01 kg in 2012.

In 2012 only, waist circumference (measured at the navel, over light or no clothing) and hip circumference (measured at the widest point over light clothing) were measured to the nearest 0.1 cm using nonflexible measuring tape. The tape was removed before taking a second measurement. If the two measurements were more than 0.5‐cm apart, a third measurement was taken and the closest two measurements were recorded. The mean difference in repeat measurements for hip and waist circumference in 2012 was 0.05 cm for both.

For women >18 years, underweight was defined as BMI < 18.5; overweight or obese as BMI ≥ 25.0; and obese as BMI ≥ 30.0 (World Health Organization, 2018). For females 15–18 years, modified BMI cut‐offs to indicate obesity among adolescents were applied, ranging from 23.94 at 15 years of age to 25.0 at 18 years of age for overweight and 28.30 at 15 years to 30.0 at 18 years for obesity (Cole, Bellizzi, Flegal, & Dietz, 2000). However, these cut‐offs did not affect the classification of any study participants as overweight or obese, compared with the adult cut‐offs of 25.0 and 30.0 (i.e., the prevalence of overweight was identical, regardless of the cut‐off applied for adolescents).

#### 2.3.3 Markers of inflammation

In both surveys, venous blood was collected and centrifuged within ~2 hr to separate plasma, which was frozen on the day of collection. Plasma concentrations of two markers of systemic inflammation, C‐reactive protein (CRP) and α1‐acid glycoprotein (AGP) were measured by ELISA (Erhardt, Estes, Pfeiffer, Biesalski, & Craft, 2004).

### 2.4 Data analysis

SAS software (Version 9.4, SAS Institute, Cary, NC) was used for data analysis. To characterize SES, factor analysis (PROC FACTOR) was used to combine variables related to household possessions; occupation, employment, and education of the index woman and household head; house construction materials; and sanitation facilities. The continuous score generated from the primary factor was then categorized into quintiles. SES scores were calculated separately for the 2009 and 2012 surveys.

Survey weights were applied at the stratum (North, South, and Yaoundé/Douala**)** and cluster level to account for the stratified design (i.e., different population sizes of each stratum) and nonresponse and exclusion of pregnant women, respectively.

Differences in participant characteristics among survey strata in 2009 and between participants in the 2009 and 2012 surveys in Yaoundé/Douala were compared using survey linear regression analysis (SAS PROC SURVEYREG) for continuous variables and chi‐square analysis for categorical variables (Rao‐Scott Chi‐square; SAS PROC SURVEYFREQ). The distribution of continuous outcome variables was examined for adherence to a normal distribution (Shapiro‐Wilkes W ≥ 0.97), and variables were transformed as necessary prior to regression analysis, and residuals were examined to confirm normality.

To examine factors associated with overweight and obesity, logistic regression (SAS PROC SURVEYLOGISTIC) was applied, with the primary outcome of overweight and obesity, defined as BMI ≥ 25.0. We did not examine overweight and obesity separately because of the small number of obesity cases in some subgroups. The risk factors examined were stratum (in the national model only), urban or rural location (national, South, and North only), household SES, household ownership of a car or TV, the woman's educational attainment and occupation, total weekly hours spent watching TV (2009 only), and any consumption in the previous week of sweetened beverages, sweets, biscuits, or beignets. We first examined the relationship between the outcomes and each individual risk factor. Second, we used multivariable logistic regression to examine independent risk factors for each outcome. Logistic regression models were developed by nonautomated backward stepwise regression that first included all potential risk factors in the model and then sequentially removed variables that were not statistically significant (*P* > 0.05). Due to substantial clustering of risk factors by stratum, we conducted the analysis at the national level and separately for each stratum.

Relationships among BMI and markers of inflammation and between BMI and waist circumference and waist‐to‐hip ratio (in the 2012 survey only) were assessed by linear regression analysis (SAS PROC SURVEYREG). Outcome variables were transformed as necessary, and residuals were examined for normality. Higher order terms were examined to confirm linearity.

## 3 RESULTS

### 3.1 Participant characteristics and prevalence of overweight and obesity

A total of 1,002 households participated in the national 2009 survey (12) and 333 households participated in the regional 2012 survey (13). We limited the analysis to *n* = 932 (2009) and *n* = 319 (2012) women for whom anthropometric data were available and excluded those who were pregnant (2009: 96 nationally; 2012: 49 Yaoundé/Douala) or for whom information on pregnancy status was unavailable (2009: 132 nationally; 2012: 27 Yaoundé/Douala). Women for whom information on pregnancy status was collected did not differ from women with missing data in age (2009: *P* = 0.32; 2012: *P* = 0.25) or BMI (2009: *P* = 0.11; 2012: *P* = 0.41).

On average, women in the North were younger than women in the South and Yaoundé/Douala in the 2009 survey, and women in Yaoundé/Douala were slightly older in the 2012 survey compared with the 2009 survey (Table 1). Adolescent mothers (15.0–17.9 years) comprised 13% of the sample in 2009 (12% South, 15% North, 11% Yaoundé/Douala) and 5% of the sample in Yaoundé/Douala in 2012. The average age of the breastfeeding child among the 29% of women who were lactating in 2009 was 10 months (median: 9.9 months; range: 1.0 to 24.0 months). In 2012, in Yaoundé/Douala, 32% were lactating, and average age of their children was 6.7 months (median 5.6 months; range: 1.3 to 22.5 months). In 2012, mean parity was 2.6 births (data not collected in 2009).

**Table 1:** Characteristics of nonpregnant women of reproductive age and their households in a national survey in 2009 (nationally and by geographic stratum) and in a regional survey in Yaoundé/Douala in 2012

		National (2009)	South (2009)	North (2009)	Yaoundé/Douala (2009)	Yaoundé/Douala (2012)
*N*		704	268	236	200	243
Caregiver age, year		27.1 [26.5, 27.8]	27.9 [26.7, 29.1]_a_	25.9 [24.8, 27.0]_b_	27.3 [26.3, 28.2]_a_	29.5 [28.5, 30.5]^a^
Caregiver lactating, %		29.4 [25.9, 33.0]	35.8 [29.8, 41.8]_a_	27.0 [20.7, 33.3]_b_	19.5 [13.5, 25.4]_b_	31.9 [26.6, 37.1]^a^
Weight, kg		61.3 [60.0, 62.7]	63.0 [60.8, 65.2]_b_	55.1 [52.6, 57.5]_c_	67.2 [65.0, 69.4]_a_	70.1 [67.2, 73.0]
Height, m		1.60 [1.60, 1.61]	1.60 [1.59, 1.61]	1.61 [1.60, 1.62]	1.61 [1.60, 1.62]	1.61 [1.61, 1.62]
Short stature (height < 1.45 m, %)		0.4 [0, 0.9]	0.5 [0, 1.5]	0.3 [0, 1.0]	0.4 [0, 1.1]	0
BMI, kg/m^2^		23.8 [23.4, 24.2]	24.6 [23.9, 25.3]_b_	21.1 [20.4, 21.9]_c_	26.0 [25.2, 26.8]_a_	26.9 [25.8, 28.0]
	BMI < 18.5, %	7.8 [5.3, 10.2]	2.1 [0.2, 3.9]_b_	19.3 [12.1, 26.5]_a_	2.5 [0.2, 4.8]_b_	4.7 [1.6, 7.7]
	Overweight or obese^b^, %	32.4 [28.1, 36.7]	40.2 [32.7, 47.8]_a_	10.6 [5.1, 16.1]_b_	48.7 [39.4, 58.0]_a_	55.5 [46.9, 64.1]
	Obese^b^, %	10.7 [8.3, 13.1]	11.5 [7.3, 15.7]_b_	3.4 [0.1, 6.7]_c_	20.1 [14.7, 25.5]_a_	24.1 [16.6, 31.6]

*Note*. Values represent mean or percent (95% CI). Values with different letter superscripts are significantly different (*P* < 0.05) among the three strata in the 2009 survey using survey linear or logistic regression procedures, as appropriate, a > b > c. Controlling for age did not affect the regional differences in BMI. BMI = body mass index.

a Significantly different from the 2009 value for Yaoundé/Douala, *P* < 0.05.

b Overweight or obese was defined as BMI ≥ 25.0 and obese defined as BMI ≥ 30.0 for women ≥18 years of age (and 17 women [<2%] with missing age data). For women 15–18 years of age, adolescent‐specific cut‐offs were used (17), but these did not change the classification of individuals as overweight or obese compared with the adult cut‐offs.

In 2009, about 8% of women were underweight (BMI < 18.5) and 32% were overweight or obese (BMI ≥ 25.0) nationally, but the patterns differed widely by region. Underweight was more common in the North region (19%), and overweight and obesity were more common in the South (40% BMI ≥ 25.0) and Yaoundé/Douala (49% BMI ≥ 25.0). Regional differences in BMI were explained by differences in weight, which varied from a mean of 55.1 kg in the North to 67.2 kg in Yaoundé/Douala (*P* < 0.0001 overall), rather than height, which did not differ on average by region (*P* = 0.12 overall).

### 3.2 Change from 2009 to 2012 in prevalence of overweight/obesity in Yaoundé and Douala

Mean BMI and prevalence of BMI ≥ 25.0 tended to be greater among participants in the 2012 survey (55.5% BMI ≥ 25.0) compared with those in the 2009 survey (48.7%) in Yaoundé and Douala (Table 1), but the differences were not statistically significant (*P* = 0.16).

### 3.3 Factors associated with overweight/obesity among women

**Figure 1:**
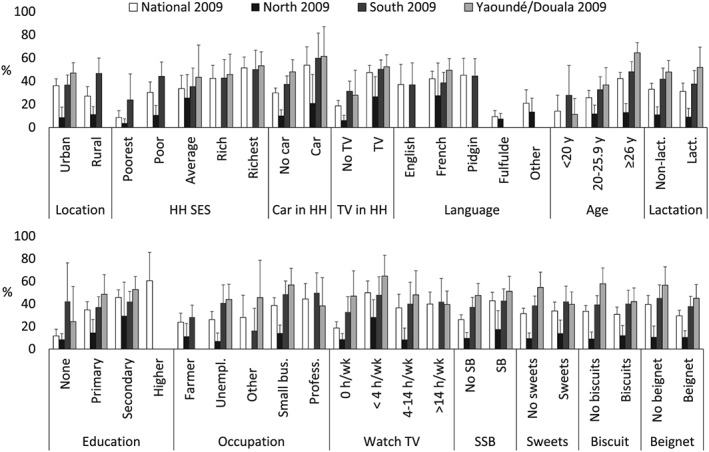
Prevalence of overweight and obesity according to potential risk factors among nonpregnant Cameroonian women, nationally and regionally in 2009, and in Yaoundé/Douala in 2012. Overweight or obese was defined as body mass index ≥ 25.0 for women ≥18 years of age (and for 17 women [<2%] with missing age data). Categories with *n* < 10 individuals were excluded from the figure (South: higher education; Fulfulde or other language. North: >14 hr/week of TV watching; professional or other occupation; higher education; English or pidgin language; rich or richest SES quintile. Yaoundé/Douala: Higher education; poorest, poor, or average SES quintile; other, pidgin, Fulfulde, or English language). HH = household; Lact = lactating; SSB = sugar‐sweetened beverage; SES = socio‐economic status

In bivariate comparisons, maternal age was the most consistent factor associated with increased odds of overweight/obesity (Figure 1; Table 2; Table S1). The mean BMI and prevalence of overweight did not differ among women who were or were not currently lactating (nationally, within geographic stratum, and within socio‐economic quintiles). However, among lactating women, BMI was negatively correlated with the infant's age (Spearman's *r* = −0.13, *P* = 0.02). Nationally, the prevalence of overweight and obesity varied by SES, from 8.6% in the lowest quintile to 51.5% in the highest quintile. Within the geographic strata, this relationship only remained significant in the North.

**Table 2:** Household and individual characteristics of nonpregnant women of reproductive age and their households in the 2009 survey (nationally and by region) and in the 2012 survey in Yaoundé/Douala

		National (2009)	South (2009)	North (2009)	Yaoundé/Douala (2009)	Yaoundé/Douala (2012)
*N*		704	268	236	200	243
Urban^a^, *n* (%)		425 (58.5)	166 (63.3)^b^_b_	62 (26.7)_c_	197 (96.7)_a_	233 (96.7)
Individuals in household^b^, *n*		7.7 [7.3–8.0]	7.4 [6.9–7.9]_b_	8.8 [8.1–9.4]_a_	6.4 [5.8–7.0]_c_	6.5 [6.1–6.9]
Household SES^a^, ^c^, *n* (%)						
	Lowest	144 (20.8)	28 (11.0)	116 (47.4)	0	46 (19.7)
	Low	145 (23.5)	78 (30.1)	67 (29.2)	0	53 (22.1)
	Average	137 (21.7)	71 (26.9)	40 (19.0)	26 (14.4)	47 (19.5)
	High	121 (17.0)	55 (21.2)	7 (2.7)	59 (30.7)	48 (20.1)
	Highest	138 (17.0)	28 (10.8)	4 (1.7)	106 (54.8)	46 (18.5)
Car in household^b^, %		7.6 [5.1–10.2]	9.4 [4.8–13.9]_a_	3.8 [1.3–6.3]_b_	9.8 [3.8–15.8]_a_	10.4 [6.3–14.6]
TV in household^b^, %		46.8 [40.9–52.7]	46.0 [35.1–56.9]_b_	21.8 [11.9–31.7]_c_	88.8 [83.0–94.5]_a_	92.2 [88.5–95.9]
Language of interview^a^, *n* (%)						
	English	38 (6.1)	34 (12.4)	1 (0.4)	3 (1.4)	13 (5.7)
	French	368 (50.9)	160 (60.7)	24 (10.2)	184 (91.9)	228 (94.3)
	Pidgin	66 (10.7)	64 (22.9)	0	2 (0.7)	0
	Fulfulde	174 (24.4)	2 (0.8)	170 (72.8)	2 (1.5)	0
	Other or missing	58 (7.8)	8 (3.2)	41 (16.5)	9 (4.6)	0
Educational attainment^a^, ^d^, *n* (%)						
	None	187 (27.7)	11 (4.8)	170 (74.5)	6 (3.6)	11 (4.7)
	Primary	253 (38.4)	142 (54.1)	52 (21.2)	59 (31.2)	75 (30.3)
	Secondary	229 (32.2)	102 (39.3)	11 (4.3)	116 (61.1)	135 (56.7)
	Higher	13 (1.7)	5 (1.8)	0 (0)	8 (4.1)	20 (8.3)
Occupation^a^, ^c^, *n* (%)						
	Professional/technical/managerial	44 (6.4)	25 (9.5)	2 (0.9)	17 (8.4)	35 (14.2)
	Farmer	104 (18.1)	75 (28.7)	29 (14.5)	0 (0)	1 (0.5)
	Small business/trader	297 (43.0)	105 (40.0)	108 (44.6)	84 (47.1)	74 (30.9)
	Other	32 (4.5)	12 (4.7)	3 (1.0)	17 (9.5)	19 (8.2)
	Unemployed	206 (28.1)	44 (17.1)	92 (39.0)	70 (35.0)	112 (46.2)
Total time watching TV in the past week^a^, *n* (%)						
	0 hr	285 (52.7)	72 (43.6)	185 (80.7)	28 (16.3)	—
	<4 hr	88 (16.3)	36 (20.6)	18 (7.6)	34 (25.0)	—
	4–14 hr	104 (18.2)	37 (22.1)	21 (9.0)	46 (28.8)	—
	>14 hr	81 (12.8)	21 (13.7)	7 (2.7)	53 (29.9)	—
Consumed sweetened beverages in past week^b^, %		36.6 [31.7, 41.4]	45.9 [36.4, 55.3]_a_	11.8 [7.1, 16.4]_b_	55.7 [46.3, 65.0]_a_	52.0 [44.4, 59.6]
Consumed sweets in past week^b^, %		31.5 [27.6, 35.4]	34.4 [27.8, 41.1]	26.2 [20.0, 32.8]	33.5 [27.1, 39.8]	31.6 [24.3, 39.0]
Consumed biscuit in past week^b^, %		47.8 [43.5, 52.1]	43.5 [36.9, 50.0]	50.9 [42.6, 59.2]	52.4 [44.2, 60.6]	41.0 [34.3, 57.6]
Consumed beignet in past week^b^, %		72.3 [67.8, 76.8]	73.6 [66.0, 81.2]_a_	77.9 [71.3, 84.5]_a_	60.6 [50.7, 70.5]_b_	52.1 [45.7, 58.5]

*Note*. SES: socio‐economic status.

a Values are *n* (weighted %). Values with different letter superscripts are significantly different (*P* < 0.05) among the three strata in the 2009 survey using survey logistic regression procedures, a > b > c.

b Values are mean or % [95% CI]. Values with different letter superscripts are significantly different (*P* < 0.05) among the three strata in the 2009 survey using survey linear regression procedures, a > b > c.

c Socio‐economic status scores were calculated separately for 2009 and 2012 surveys so are not directly comparable.

d Patterns of caregiver education and occupation differ by region, Rao–Scott chi‐square statistic, *P* < 0.001.

Household TV ownership was associated with greater prevalence of obesity in all survey strata in 2009, but not in Yaoundé/Douala in 2012. Hours spent watching TV over the previous week was associated with overweight nationally but not within strata. Presence of a TV in the household was significantly related to SES quintile (*P* < 0.001): 7% of households in the poorest quintile had a TV, compared with 97% of the households in the richest quintile. In general, prevalence of overweight was lowest among women who were farmers (<30%) compared with women employed in small business or professional positions (>35%), except in the North, where the prevalence of overweight was <30% in all subgroups.

Nationally, the prevalence of overweight was greater among women who consumed sweetened beverages in the past week compared with those who did not consume sweetened beverages in the past week (43% vs. 26%, *P* < 0.0001), but this difference did not persist within geographic strata. Consumption of sweets or biscuits in the past week was not associated with prevalence of overweight, but prevalence of overweight was lower among women who consumed beignets in the past week (29% vs. 40%, *P* = 0.028). Sweetened beverage consumption was strongly related to SES, with the proportion of women who consumed sweetened beverages in the previous week increasing monotonically from 12% in the lowest SES quintile to 67% in the highest SES quintile (overall Rao Scot Chi‐square *P* < 0.0001). The proportion of women who consumed beignet in the previous week was 72–79% in the three lowest SES quintiles and 63–66% in the highest two SES quintiles (Rao Scott Chi‐square *P* = 0.03 overall). Consumption of sweets or biscuits in the past week was not associated with SES quintile (*P* > 0.4).

In multivariable models, factors that were independently associated with greater prevalence of overweight were residence in Yaoundé/Douala or in an urban area, older age, and household TV ownership (Table 3). Household TV ownership was also significantly associated with overweight within all three strata in 2009 (although the association was marginal the North region, *P* = 0.066). Being older was also independently associated with overweight in the North and Yaoundé/Douala in 2009 and was the only significant predictor of overweight in Yaoundé/Douala in 2012.

**Table 3:** Odds ratios [95% CI] for overweight or obesity from multivariable logistic regression analysis of potential risk factors among nonpregnant Cameroonian women, nationally and regionally in 2009, and in Yaoundé/Douala in 2012

		National (2009)	South (2009)	North (2009)	Yaoundé/Douala (2009)	Yaoundé/Douala (2012)
	*N*	677	250	222	179	243
Variable	Levels					
Milieu (referent = urban)						
	Rural	0.52 [0.30, 0.92]	—	—	—	—
Stratum (referent = Yaoundé/Douala)						
	South	0.82 [0.49, 1.35]	—	—	—	—
	North	0.14 [0.06, 0.33]	—	—	—	—
Age (referent = ≥26 years)						
	20–25.9 years	0.50 [0.34, 0.75]	—	0.65 [0.26, 1.64]	0.27 [0.14, 0.51]	0.43 [0.25, 0.72]
	<20 years	0.27 [0.11, 0.68]	—	<0.01 [<0.01, <0.01]	0.07 [0.01, 0.33]	0.38 [0.12, 1.20]
Household TV ownership (referent = household owns TV)						
	No TV in household	0.35 [0.24, 0.51]	0.46 [0.31, 0.69]	—	0.31 [0.11, 0.92]	—
SES quintile (referent = average)						
	Low	—	—	0.30 [0.10, 0.96]	—	—
	Lowest	—	—	0.08 [0.02, 0.34]	—	—

*Note*. Overweight or obese was defined as BMI ≥ 25.0 for women ≥18 years of age (and 17 women [<2%] with missing age data). For women 15–18 years of age, adolescent‐specific cut‐offs were used (17), but these did not change the classification of individuals as overweight or obese compared with the adult cut‐offs. Subgroups with *n* < 10 individuals were excluded from models. Nonsignificant variables (*P* > 0.05) were removed using a stepwise backward regression method. In the South (2009), rural location was removed at *P* = 0.07 (OR for rural location = 0.57, 95% CI [0.31, 1.06]). In the North (2009), household TV ownership was removed at *P* = 0.07 (OR for no TV in household = 0.41, 95% CI [0.16, 1.06]). In Yaoundé/Douala (2009), biscuit consumption in the past week was removed at *P* = 0.051 (OR for no biscuits in last week = 2.05, 95% CI [1.00, 4.19]). All other covariates removed at *P* > 0.1 (covariates not shown: household car ownership, education, lactation status, weekly hours of TV watched, and consumption of sweetened beverages, sweets, or beignets in the past week). SES: socio‐economic status; BMI: body mass index.

In 2012 in Yaoundé/Douala, parity was associated with increased odds of being overweight (OR = 1.27 (1.06–1.52), *P* = 0.01), but the relationship was no longer significant after controlling for age (OR = 1.09 (0.86–1.38), *P* = 0.47).

### 3.4 Associations between BMI and markers of abdominal obesity and inflammation

In Yaoundé/Douala in 2012, BMI was positively associated with waist circumference, waist‐to‐hip ratio, and waist‐to‐height ratio (Ln [Waist circumference]: Beta [95% CI] for lnBMI = 0.68 [0.65, 0.71], *R* ^2^ = 0.89; waist‐to‐hip ratio: lnBMI = 0.14 [0.12, 0.16], *R* ^2^ = 0.29; inverse [waist‐to‐height ratio]: lnBMI = −1.19 [−1.25, −1.13], *R* ^2^ = 0.87; *P* < 0.0001 for all; *n* = 243; Figures S1and S2; Table 4). In 2009, greater BMI predicted greater plasma CRP and AGP concentrations in the North (lnCRP: Beta [95% CI] for lnBMI = 1.36 [0.35, 2.36], *R* ^2^ = 0.02, *P* = 0.01; lnAGP: lnBMI = 0.19 [0.06, 0.33], *R* ^2^ = 0.03, *P* = 0.05) and in Yaoundé/Douala (lnCRP: lnBMI = 2.70 [1.46, 3.93], *R* ^2^ = 0.11, *P* < 0.0001; lnAGP: lnBMI = 0.30 [0.16, 0.44]), *R* ^2^ = 0.07, *P* < 0.0001). In the South region, the relationships between the natural logarithms of CRP and BMI and AGP and BMI were both nonlinear (with or without natural logarithm transformation of BMI): CRP and AGP concentrations were greater at both low and high BMI levels. In Yaoundé/Douala in 2012, BMI was also positively associated with plasma CRP concentration (lnCRP: Beta [95% CI] for lnBMI = 2.15 [1.29, 3.00], *R* ^2^ = 0.11, *P* < 0.0001) and plasma AGP concentration (lnBMI = 0.43 ± [0.28, 0.58], *R* ^2^ = 0.10, *P* < 0.0001).

**Table 4:** Waist circumference, waist:hip ratio, and waist:height ratios among nonpregnant women 15–49 years of age in Yaoundé and Douala, Cameroon (*n* = 243)

	Mean or % [95% CI]
Waist circumference, cm	90.0 [87.5, 92.5]
Hip circumference	105.3 [103.2, 107.4]
Waist:hip ratio	0.85 [0.84, 0.86]
Waist:height ratio	0.56 [0.54, 0.58]
Waist circumference > 80 cm, %	73.0 [65.0, 80.9]
Waist circumference > 88 cm, %	46.0 [37.0, 54.6]
Waist:hip ratio > 0.85, %	48.4 [40.4, 56.4]
Waist:height ratio > 0.5, %	71.3 [64.2, 78.4]

## 4 DISCUSSION

This study presents estimates on the prevalence and geographic distribution of overweight and obesity among women in Cameroon, a transitioning country, and examines demographic and behavioural risk factors. Though causal inferences cannot be made between individual behaviours/characteristics and overweight/obesity, the findings of this cross‐sectional national study are useful to identify subgroups at greatest risk for overweight and associated health consequences in Cameroon. Wide regional variation exists in the prevalence of overweight and obesity in Cameroon, with a large burden among women in the South region and in Yaoundé and Douala, whereas underweight among women remains prevalent in the North region. In addition to urban residence, overweight/obesity was independently associated with older maternal age and ownership of a TV.

TV ownership could be an indicator of physical inactivity but also may serve as a proxy for SES or preferred leisure activities. An exploratory analysis suggested that both pathways may be present. When SES score and household TV ownership were introduced into the same model predicting women's BMI (nationally in 2009), both predictors were significantly associated with BMI. However, their coefficients were reduced, compared with those from the bivariate relationships between each predictor and maternal BMI. The proportion of households with a TV ranged from 22% in the North to around 90% in Yaoundé/Douala. The corresponding population attributable fraction for overweight and obesity among women was 42–44%, with or without adjusting for region. Although causality cannot be attributed, presence of a TV in a household was a strong predictor of overweight in this study and may be an important avenue for targeting households or delivering messages related to prevention and management of overweight.

Sweetened beverage consumption was associated with overweight nationally, but not within each of the three survey strata. The overall association may reflect the contribution of sweetened beverages to total energy intake but also an association between sweetened beverage consumption and SES. The lack of association within strata may reflect reduced statistical power due to the lower sample size and/or lower variation in sweetened beverage consumption and overweight within strata (compared with between strata). Consumption of beignets was associated with lower prevalence of overweight, but this relationship was not significant in multivariable models and may reflect confounding by SES since there was a trend toward greater consumption of beignets among women in the three lowest SES quintiles. Other dietary variables (consumption of sweets and biscuits) were not associated with overweight, possibly because the FFQ was designed for another purpose (estimating intake of selected fortifiable foods) rather than focusing on processed, sweetened snack foods specifically. However, another cross‐sectional study that administered a FFQ among adults of African origin from Cameroon, Jamaica, and the United Kingdom did not find any dietary variables, except for higher protein intake, to be related to overweight or obesity in Cameroon women (Jackson et al., 2007).

The observation that mean BMI and prevalence of overweight did not differ among lactating women compared with nonlactating women may be explained by the wide age range of infants of lactating women in this sample (in 2009: 1 to 24 months of age; mean of 10 months). In the early postpartum period, lactating women are likely to have higher BMI than nonlactating women, reflecting weight gain during pregnancy. However, prolonged lactation may contribute to reduced risk of overweight; our observation of a negative correlation between BMI and infant age among lactating women supports this. The contribution of postpartum weight retention to overweight among women and the potential role of breastfeeding support to weight management in this setting should be explored further. Among women in Yaoundé/Douala, higher BMI was associated with greater waist circumference and greater waist‐to‐hip ratio, similar to a previous study among adults in Yaoundé (Pasquet, Temgoua, Melaman‐Sego, Froment, & Rikong‐Adié, 2003). These findings suggest that overweight in this context may be associated with increased cardiometabolic risk. We measured waist circumference at the navel, rather than midway between the lowest rib and iliac crest, which may introduce error into the measures of waist circumference and waist‐to‐hip ratio. Nevertheless, this protocol predicted abdominal obesity as measured by dual energy X‐ray absorptiometry (DXA) among adults (Pimenta et al., 2016). BMI was also associated with elevated concentrations of CRP and AGP, which are markers of systemic inflammation used to indicate risk of cardiovascular disease in high‐income countries. Because the survey was designed with a focus on micronutrient deficiencies, we did not collect information on physical activity, body composition, or specific biomarkers of cardiometabolic risk. However, the association of BMI with abdominal obesity and the existing data on health behaviours (such as consumption of sweetened beverages) suggest a pattern consistent with risk of developing noncommunicable disease, which has been observed elsewhere in Africa (Tugendhalf et al., 2016), particularly in urban areas.

The findings reported here are consistent with the growing base of evidence documenting a high and increasing prevalence of overweight and obesity and comorbidities such as diabetes in Africa. In Cameroon, the current prevalence estimates of overweight and obesity (BMI ≥ 25.0) among women are similar to previous studies in the country. Specifically, the national figure reported here of 32% (95% CI [28.1, 36.7]) BMI ≥ 25.0 among women 15–49 years of age who were primary caregivers for a child 12–59 months of age concurs well with the estimate of about 30%, as reported in the 2011 Demographic and National Survey for nonpregnant Cameroon women, ages 15–49 years (Jones et al., 2016). A community‐based sample from an urban district of Yaoundé had an overweight/obesity prevalence of 54.9% in 2000 among nonpregnant women 25 years and older (Fezeu et al., 2010), which matches well with this study's estimate of 48.7 to 55.5%, the 2009 to 2012 Yaoundé/Doula figures for nonpregnant women ages 15–49 years. The earlier study also reported the estimate of abdominal obesity to be 67% in 2000 (waist circumference ≥ 80 cm), compared with 73% reported here in 2009–2012 for Yaoundé/Doula women (Fezeu et al., 2010).

Though mean BMI increased from 26.0 to 26.8 in Yaoundé/Douala from 2009 to 2012, this difference was not significant and appears inconsistent with other analyses that report increasing trends in BMI among women in urban areas of sub‐Saharan Africa (NCD Risk Factor Collaboration (NCD‐RisC), 2016). The lack of statistical significance is likely the result of inadequate sample size; at least 600 individuals per group would have been required to detect a change in mean BMI of 0.8 units, assuming *SD* = 5 (excluding the design effect). Also, the time period between surveys (3 years) was relatively short.

In this study, the prevalence of overweight was greatest in the stratum composed of the two largest cities, and urban residence remained significantly related to overweight in multivariable models. Other African studies have noted greater risk of overweight in urban areas (Jones et al., 2016; Kandala & Stranges, 2014). With increasing urbanization in Africa, urban food markets and supply chains have expanded and transformed to provide a wider array of Western‐style, energy‐dense products that are high in added sugars, salt, fat, and protein (Haggblade et al., 2016). Concurrent increases in per capita income have enabled greater consumer demand for these products and led to dietary changes, physical inactivity, and other lifestyle behaviours that increase risk of obesity and noncommunicable, chronic diseases. Importantly, BMI in this study was associated with indicators of abdominal obesity and inflammation among women in Yaoundé and Douala, suggesting that the changes in obesity prevalence will have important adverse health consequences.

Haggblade et al. provides an excellent overview of potential solutions to change the trajectory of the nutrition transition in sub‐Sahara Africa (Haggblade et al., 2016). In African countries (such as Uganda) at earlier stages of this nutrition transition from undernutrition to overnutrition, public health systems have largely continued to focus their limited resources on undernutrition. In contrast, in South Africa, where more than 40% of adults are overweight or obese, national strategies have attempted to address some issues related to affordability and availability of healthy foods but the impact remains to be evaluated.

Faced with an underfunded public health system, many African countries may move forward first on supply‐side changes/interventions in the food system to reverse the growing obesity and chronic disease trends (Haggblade et al., 2016). For example, supply chain interventions can promote the development and marketing of healthier food products, based on indigenous foods, and expand urban horticulture. Meanwhile, the public health sector, working through its existing and scaled‐up maternal and child health programs, can address emerging obesity and chronic disease prevention through raising awareness and education. More interventions should be launched in schools to educate and enable children to make healthy food choices in that setting. It will also be important to train the next generation of food industry professionals in the potential effects of food technology and agribusiness policies on public health and human nutrition.

In Cameroon, policy makers could consider similar approaches to address supply‐side and consumer demand issues. For example, development of dietary guidelines to facilitate nutrition education activities (focused on both overnutrition and undernutrition) through different channels (in schools, at work, through mass media, etc.) may also help create a demand for the healthier products. The prevalence information provided in this study and other studies can be used to develop targeted social marketing campaigns to promote healthy food choices and physically active lifestyles. Such efforts may be most effective if adapted to the distinct environments and dietary habits of the geographic regions and may focus initially on the urban populations where prevalence of overweight is highest. A WHO STEPS survey conducted in Cameroon in 2003 reported that fruits were consumed on average 2.8 days per week and vegetables only 3.2 days per week among adults in urban areas, and that 44.3% of participating adults had low levels of physical activity (WHO STEPS chronic disease risk factor surveillance, 2003). Though prevalence of hypertension and diabetes have increased in Cameroon, larger body size may still be viewed positively among some ethnic groups (Cohen, Boetsch, Palstra, & Pasquet, 2013). More than 90% of urban adults in Cameroon reported sedentary activity during their leisure time, and many younger adults are also sedentary at work (Kengne, Awah, Fezeu, & Mbanya, 2007). To launch social marketing campaigns, more qualitative and quantitative research needs to explore social norms and perceptions about healthy diets, health risks of physical inactivity (including too much TV/screen time), culturally acceptable leisure time physical activities, and the relationship of body weight to health.

## 5 CONCLUSION

Overweight and obesity are common among women in Cameroon, particularly in the largest cities of Yaoundé and Douala and in urban areas generally. Overweight was more common among households with greater socio‐economic status and among older women and women with more education. Household and individual characteristics such as TV ownership and sweetened beverage consumption were also associated with prevalence of overweight. Because of the study design, we cannot determine whether the observed associations between overweight and behaviours such as TV ownership are causal in nature. However, these observations are valuable in identifying subgroups of the population who are at highest risk for overweight and should be targeted for related interventions. Geographic region, in particular, is likely to be an important consideration for program planning. BMI was associated with markers of abdominal obesity and inflammation, suggesting an increased risk of adverse health consequences if trends toward increased BMI continue.

Finally, although our analysis focused on predictors of overweight, it is important to note that underweight was common among women in the northern regions (19%), in addition to anaemia, stunting, and micronutrient deficiencies. Efforts to address undernutrition and micronutrient malnutrition should continue, but evolve toward prevention of malnutrition in all its forms (overnutrition and undernutrition) throughout the life cycle to address the double burden of malnutrition.

## CONFLICTS OF INTEREST

KHB is an employee of the Bill & Melinda Gates Foundation. All other authors declare that they have no conflicts of interest.

## CONTRIBUTIONS

RES, MN, AN, and KHB designed the original surveys; RES, MN, AN, AF, and AT supervised data collection; AF supervised data entry and cleaning; RES, KHB, and LK conceived the present analysis; RES analysed the data; RES and LK wrote the paper; all authors contributed to revisions of the paper and approved the final content.

## Supporting information


**Supplemental Table 1.** Prevalence of overweight and obesity (BMI > 25.0) according to potential risk factors among non‐pregnant Cameroonian women, nationally and regionally in 2009 (*n* = 704 nationally; 268 South, 236 North, 200 Yaoundé/Douala), and in Yaoundé/Douala in 2012 (*n* = 243). *Values are % (95% CI). P values represent Rao‐Scott Chi‐Square statistics for comparison among potential risk factors within each geographic region. Values for potential risk factors with different letter superscripts are statistically significantly different (*P* < 0.05) using logistic regression (SAS proc surveylogistic).
**Supplemental Figure 1.** Relationships between waist circumference and BMI among women in Yaoundé and Douala, Cameroon (*n* = 243). The regression coefficient was (beta (95% CI) = 0.68 (0.65 to 0.71), R^2^ = 0.89, *P* < 0.0001, for ln (waist circumference and ln (BMI)).
**Supplemental Figure 2.** Relationships between waist: hip ratio, waist: height ratio and BMI among women in Yaoundé and Douala, Cameroon (*n* = 243). The regression coefficient for waist: hip ratio was (beta (95% CI) = 0.14 (0.12 to 0.16), R^2^ = 0.29, *P* < 0.0001, for WHR and ln (BMI). The regression coefficient for waist: height ratio was beta (95% CI) = −1.19 (−1.25 to −1.13), R^2^ = 0.87, P < 0.0001, for inverse of waist: height ratio and ln (BMI).
