# Supplementary material for: Prevalence and predictors of overweight and obesity among Cameroonian women in a national survey and relationships with waist circumference and inflammation in Yaoundé and Douala
Source: Matern Child Nutr. 2018 Jul 26;14(4):e12648. doi: 10.1111/mcn.12648 (PMC6174999; doi:10.1111/mcn.12648)
Supplement: Supplementary file 1 — Supplemental Table 1. Prevalence of overweight and obesity (BMI > 25.0) according to potential risk factors among non‐pregnant Cameroonian women, nationally and regionally in 2009 (n = 704 nationally; 268 South, 236 North, 200 Yaoundé/Douala), and in Yaoundé/Douala in 2012 (n = 243). *Values are % (95% CI). P values represent Rao‐Scott Chi‐Square statistics for comparison among potential risk factors within each geographic region. Values for potential risk factors with different letter superscripts are statistically significantly different (P < 0.05) using logistic regression (SAS proc surveylogistic). Supplemental Figure 1. Relationships between waist circumference and BMI among women in Yaoundé and Douala, Cameroon (n = 243). The regression coefficient was (beta (95% CI) = 0.68 (0.65 to 0.71), R2 = 0.89, P < 0.0001, for ln (waist circumference and ln (BMI)). Supplemental Figure 2. Relationships between waist: hip ratio, waist: height ratio and BMI among women in Yaoundé and Douala, Cameroon (n = 243). The regression coefficient for waist: hip ratio was (beta (95% CI) = 0.14 (0.12 to 0.16), R2 = 0.29, P < 0.0001, for WHR and ln (BMI). The regression coefficient for waist: height ratio was beta (95% CI) = −1.19 (−1.25 to −1.13), R2 = 0.87, P < 0.0001, for inverse of waist: height ratio and ln (BMI). [file MCN-14-e12648-s001.docx]

**SUPPLEMENTARY APPENDIX**

**Supplemental Table 1.** Prevalence of overweight and obesity (BMI > 25.0) according to potential risk factors among non-pregnant Cameroonian women, nationally and regionally in 2009 (n=704 nationally; 268 South, 236 North, 200 Yaoundé/Douala), and in Yaoundé/Douala in 2012 (n=243).

|  |  | National  (2009) | South  (2009) | North  (2009) | Yaoundé/Douala  (2009) | Yaoundé/Douala (2012) |
| --- | --- | --- | --- | --- | --- | --- |
| Household location |  |  |  |  |  |  |
|  | Urban | 36.1 (30.2-42.0) | 36.6 (27.9-45.3) | 8.7 (0-17.7) | 47.0 (38.0-55.9) | 55.0 (46.1-63.9) |
|  | Rural | 27.1 (19.0-35.3) | 46.6 (33.2-59.9) | 11.3 (4.5-18.0) | -- | -- |
|  | P (Wald) | 0.11 | 0.20 | 0.66 | -- | -- |
| Household SES quintile |  |  |  |  |  |  |
|  | Poorest | 8.6 (2.7-14.5)c | 23.8 (1.3-46.2) | 3.7 (0-7.5)b | - | 56.9 (41.8-72.1) |
|  | Poor | 30.3 (21.2-39.3)b | 44.1 (31.7-56.5) | 10.7 (2.4-19.0)ab | - | 54.9 (39.0-70.9) |
|  | Average | 33.6 (22.2-45.0)b | 35.3 (19.2-51.3) | 25.7 (5.7-45.7)a | 43.3 (15.5-71.2) | 51.4 (35.0-67.8) |
|  | Rich | 42.4 (31.1-53.6)ab | 42.7 (26.8-58.6) | -- (n=7) | 45.7 (28.1-63.3) | 52.4 (36.5-68.3) |
|  | Richest | 51.5 (42.2-60.9)a | 50.0 (33.2-66.8) | -- (n=4) | 53.3 (41.3-65.3) | 59.7 (45.8-73.6) |
|  | P | < 0.0001 | 0.43 | 0.01 | 0.65 | 0.93 |
| Car in household |  |  |  |  |  |  |
|  | No car | 29.9 (25.8-34.1)b | 37.2 (29.8-44.5)b | 10.2 (5.2-15.3) | 48.0 (37.4-58.5) | 54.6 (45.5-63.7) |
|  | Car | 53.8 (38.0-69.6)a | 59.8 (38.1-81.6)a | 21.0 (0-49.6) | 61.5 (35.9-87.1) | 60.6 (44.8-76.4) |
|  | P | 0.0019 | 0.039 | 0.25 | 0.31 | 0.28 |
| TV in household |  |  |  |  |  |  |
|  | No TV | 18.7 (14.0-23.4)b | 31.2 (22.3-40.0)b | 6.2 (1.9-10.5)b | 27.8 (6.1-49.4) | 42.9 (17.3-68.6) |
|  | TV | 47.4 (41.2-53.6)a | 50.2 (42.1-58.3)a | 26.8 (9.7-43.8)a | 52.4 (42.1-62.7) | 56.2 (47.7-64.8) |
|  | P | <0.0001 | <0.0001 | 0.0009 | 0.050 | 0.46 |
| Language of interview |  |  |  |  |  |  |
|  | English | 37.1 (19.6-54.6)bc | 36.8 (17.8-55.8)a | -- (n=1) | -- (n=3) | 47.8 (18.2-77.3) |
|  | French | 42.0 (35.4-48.5)c | 38.5 (29.4-47.7)a | 27.7 (0-55.9) | 49.4 (39.2-59.5) | 55.6 (46.8-64.5) |
|  | Pidgin | 45.1 (30.5-60.0)c | 44.4 (29.2-59.6)a | -- | -- (n=2) | -- |
|  | Fulfulde | 9.3 (4.0-14.6)a | -- (n=2) | 7.5 (2.9-12.2) | -- (n=1) | -- |
|  | Other/missing | 20.9 (9.3-32.6)b | -- (n=7) | 13.6 (1.9-25.3) | -- (n=7) | -- |
|  | P | <0.0001 | 0.72 | 0.16 | --- | 0.59 |
| Age |  |  |  |  |  |  |
|  | <20 y | 14.1 (0.4-27.8)b | 27.7 (1.8-53.6) | 0b | 11.3 (0-27.5)b | 39.2 (9.7-68.7)ab |
|  | 20-25.9 y | 25.7 (19.4-32.1)b | 32.6 (21.4-43.7) | 11.9 (4.5-19.3)a | 36.7 (21.8-51.7)b | 41.9 (30.2-53.5)a |
|  | >=26 y | 42.2 (36.8-47.6)a | 48.1 (39.4-56.9) | 13.1 (5.5-20.6)a | 64.5 (55.7-73.4)a | 62.9 (53.7-72.2)b |
|  | P | <0.0001 | 0.051 | <0.0001 | <0.0001 | 0.003 |
| Physiological status |  |  |  |  |  |  |
|  | Non-lactating | 32.9 (27.7-38.1) | 41.7 (32.1-51.3) | 11.1 (4.4-17.9) | 47.9 (37.9-57.9) | 59.7 (46.7-72.8) |
|  | Lactating | 31.1 (23.9-38.4) | 37.6 (26.1-49.2) | 9.1 (1.7-16.5) | 51.8 (34.3-69.4) | 53.3 (44.9-61.7) |
|  | P | 0.69 | 0.58 | 0.66 | 0.67 | 0.25 |
| Education |  |  |  |  |  |  |
|  | None | 11.6 (5.6-17.6)c | 42.2 (8.1-76.4) | 8.5 (3.4-13.6) | 24.2 (0-62.4) | 59.4 (18.0-100) |
|  | Primary | 34.7 (27.4-41.9)b | 36.7 (27.0-46.4) | 14.5 (2.8-26.3) | 48.5 (31.1-66.0) | 58.2 (48.9-67.5) |
|  | Secondary | 45.7 (38.9-52.4)a | 42.0 (32.8-51.2) | 29.4 (0-59.7) | 52.8 (41.3-64.2) | 52.7 (41.1-64.3) |
|  | Higher | 60.6 (35.6-85.5)ab | --(n=5) | -- | -- (n=8) | 59.1 (34.3-83.9) |
|  | P | <0.0001 | 0.65 | 0.09 | 0.45 | 0.89 |
| Occupation |  |  |  |  |  |  |
|  | Farmer | 23.7 (15.6-31.9)b | 28.3 (17.7-39.0)b | 11.2 (0-22.8) | -- | -- |
|  | Unemployed | 26.0 (18.6-33.4)b | 40.9 (24.8-56.9)ab | 7.0 (0-14.5) | 43.9 (30.4-57.3) | 51.5 (42.0-61.0)b |
|  | Other | 28.0 (8.2-47.7)ab | 16.3 (0-39.6)ab | -- (n=3) | 45.6 (12.6-78.7) | 43.6 (13.9-73.4)ab |
|  | Small business | 38.6 (31.7-45.5)a | 48.6 (36.8-60.3)a | 14.1 (6.9-21.4) | 56.8 (42.1-71.4) | 69.0 (58.9-79.0)a |
|  | Professional | 44.4 (30.7-58.2)a | 49.8 (32.0-67.5)a | -- (n=3) | 38.2 (13.0-63.3) | 45.8 (28.3-63.3)b |
|  | P | 0.0025 | 0.019 | 0.29 | 0.34 | 0.004 |
| TV time category |  |  |  |  |  |  |
|  | 0 h/wk | 18.6 (13.1-24.2)b | 32.8 (19.1-46.4) | 8.6 (3.4-13.9)b | 46.9 (24.5-69.3) | -- |
|  | < 4 h/wk | 49.9 (39.5-60.4)a | 48.0 (31.9-64.1) | 28.3 (12.9-43.7)a | 64.6 (46.0-83.1) | -- |
|  | 4-14 h/wk | 36.5 (24.5-48.5)a | 40.1 (21.0-59.1) | 8.4 (0-20.3)b | 48.0 (26.6-69.4) | -- |
|  | >14 h/wk | 39.9 (29.3-50.5)a | 41.9 (21.3-62.5) | -- (n=7) | 39.5 (27.6-51.5) | -- |
|  | P | < 0.001 | 0.52 | 0.11 | 0.098 | -- |
| Sweetened beverages (SB) in past week |  |  |  |  |  |  |
|  | No SB | 26.0 (21.7-30.4) | 37.3 (28.7-45.9) | 9.7 (4.8-14.7) | 47.5 (36.7-58.2) | 53.0 (41.9-64.1) |
|  | SB | 42.8 (35.3-50.2) | 42.8 (32.3-53.3) | 17.5 (1.0-34.1) | 51.2 (38.0-64.4) | 57.2 (45.5-69.0) |
|  | P | <0.0001 | 0.33 | 0.18 | 0.56 | 0.56 |
| Sweets in past week |  |  |  |  |  |  |
|  | No sweets | 31.4 (26.7-36.2) | 38.6 (30.3-46.9) | 9.5 (4.7-14.2) | 54.6 (41.0-68.1) | 58.5 (47.2-69.7) |
|  | Sweets | 33.8 (26.0-41.6) | 42.1 (28.4-55.7) | 14.0 (2.4-25.7) | 39.6 (28.5-50.6) | 48.1 (34.6-61.7) |
|  | P | 0.58 | 0.64 | 0.30 | 0.053 | 0.24 |
| Biscuit in past week |  |  |  |  |  |  |
|  | No biscuits | 33.5 (28.4-38.7) | 39.5 (31.7-47.3) | 9.2 (3.3-15.1) | 57.8 (43.7-71.9) | 58.4 (48.7-68.1) |
|  | Biscuits | 30.7 (24.1-37.2) | 40.2 (28.3-52.0) | 12.1 (3.2-20.9) | 42.0 (29.9-54.2) | 50.6 (38.8-62.4) |
|  | P | 0.47 | 0.92 | 0.55 | 0.058 | 0.21 |
| Beignet |  |  |  |  |  |  |
|  | No beignet | 39.5 (31.4-47.6)a | 45.2 (33.5-56.9) | 10.7 (1.0-20.4) | 56.6 (40.4-72.9) | 51.2 (39.4-63.0) |
|  | Beignet | 29.4 (24.4-34.3)b | 37.9 (29.1-46.6) | 10.6 (5.0-16.3) | 44.9 (32.6-57.2) | 58.9 (49.9-67.8) |
|  | P | 0.028 | 0.27 | 0.99 | 0.23 | 0.18 |

*Values are % (95% CI). P values represent Rao-Scott Chi-Square statistics for comparison among potential risk factors within each geographic region. Values for potential risk factors with different letter superscripts are statistically significantly different (P<0.05) using logistic regression (SAS proc surveylogistic).

**Supplemental Figure 1.** Relationships between waist circumference and BMI among women in Yaoundé and Douala, Cameroon (n=243). The regression coefficient was (beta (95% CI) = 0.68 (0.65 to 0.71), R^2^ = 0.89, P<0.0001, for ln(waist circumference and ln(BMI)).

**Supplemental Figure 2.** Relationships between waist:hip ratio, waist:height ratio and BMI among women in Yaoundé and Douala, Cameroon (n=243). The regression coefficient for waist:hip ratio was (beta(95% CI) = 0.14 (0.12 to 0.16), R^2^ = 0.29, P<0.0001, for WHR and ln(BMI). The regression coefficient for waist:height ratio was beta (95% CI) = -1.19 (-1.25 to -1.13), R^2^=0.87, P<0.0001, for inverse of waist:height ratio and ln(BMI).
